# Predicting pro-environmental behavioral intention using interpretable machine learning

**DOI:** 10.1038/s41598-026-52271-7

**Published:** 2026-05-11

**Authors:** Jing Li, Meng Liu, Meng Zhang, Zhiling Wang

**Affiliations:** 1 School of Management, Tianjin University of Commerce, Tianjin, China; 2 Research Center for Management Innovation and Evaluation, Tianjin University of Commerce, Tianjin, China; 3 School of Business Administration, Hebei University of Economics and Business, Shijiazhuang, China; 4 Department of Management, Henan University of Economics and Law, Zhengzhou, China

**Keywords:** Pro-environmental behavioral intention, Machine learning, Determinant model, CGSS, Environmental sciences, Environmental social sciences, Mathematics and computing

## Abstract

This study employs machine learning techniques to investigate the determinants of pro-environmental behavioral intention (PEBI) using the survey data from the 2021 wave of the Chinese General Social Survey (CGSS). By comparing the predictive performance of nine prevalent machine learning algorithms, the results show that the Random Forest model achieves the highest predictive accuracy in predicting PEBI. Furthermore, we develop an interpretable model based on the Random Forest and Shapley additive explanations (SHAP) analysis to examine the relative importance of 20 key predictors. The findings reveal that environmental concern is the most influential factor and is positively related to individual PEBI, followed by environmental attitude, connection to nature, environmental knowledge, government performance perception, and environmental responsibility. Additionally, we also examine the heterogeneity across different subsamples and explore interaction effects between key variables. Overall, this study adds novel knowledge to environmental sustainability literature by employing machine learning methods to predict the influencing factors of PEBI.

**Supplementary Information:**

The online version contains supplementary material available at 10.1038/s41598-026-52271-7.

## Introduction

Climate change is one of the most pressing environmental challenges confronting humanity, with far-reaching consequences for ecosystems, economies, and human health^1^. Although natural environmental changes may contribute to climate variability, human activities remain the dominant driver of environmental degradation. Recent research has highlighted that individual behavioral patterns play a pivotal role in addressing environmental problems and advancing environmental sustainability^2^. Accordingly, pro-environmental behavior (PEB), as a critical force in mitigating the negative impacts of human activity on the natural environment, has attracted increasing scholarly attention in recent decades^3,4^. Drawing on the theory of planned behavior (TPB), behavioral intention is widely recognized as a key driver of actual behavior^5^. Therefore, it follows that pro-environmental behavioral intention (PEBI) can be understood as individuals’ readiness to engage in environmentally responsible actions and serves as an important precursor of PEB. Particularly in the context of the ongoing development of industrial civilization, fostering individuals’ PEBI and encouraging the adoption of concrete actions are essential for effectively addressing environmental challenges.

Significant efforts have indeed been devoted to investigating individuals’ PEBI and its influential factors. Existing studies suggest that the drivers of PEBI are multifaceted, encompassing environmental mindsets (e.g., environmental attitude, environmental concern, and environmental knowledge)^6,7^, social ecological interactions (e.g., social norm, social network, and connection to nature)^8,9^, well-being (e.g., emotion and health)^10^, as well as demographic characteristics^11^. For example, a meta-analysis conducted by Lin et al^12^. offered a comprehensive synthesis, demonstrating that PEBI is influenced by multiple dimensions of antecedents, including self-efficacy, affect, environmental mindsets, norms and values, and perception and evaluation.

While prior studies have provided valuable insights into the antecedents of PEBI, there are still two unresolved concerns in current research. First, the prior research has predominantly predicted PEBI based on single-dimensional indicators or a limited set of explanatory variables, which may fail to provide a holistic picture of PEBI. Although researchers have attempted to construct integrative frameworks to capture multiple drivers of PEBI, they have largely focused on identifying correlations among variables^12^. As a result, there remains a lack of consensus regarding which factors serve as the most critical determinants of PEBI. Second, the majority of existing studies have employed traditional regression techniques to estimate theoretical models, which are limited by assumptions of normal data distribution, multicollinearity, and linear relationships. We argue that further insights can be gleaned through the adoption of the machine learning methods. Current research has argued that machine learning offers significant methodological advantages in handling the complex nonlinear relationships of multidimensional variables in large datasets^13^. Moreover, machine learning methods tend to generate more robust results, thereby strengthening the overall credibility of these methods^14^. As such, machine learning techniques hold great promise for advancing the study of PEBI.

To address the gap identified above, this study employs machine learning to construct a prediction model for PEBI. It then investigates whether and how the 20 critical variables predict PEBI from four aspects of environmental mindset, social-ecological interaction, well-being, and demographic characteristics. By doing so, this study makes three primary contributions. First, this study develops an integrative predictive framework for PEBI by systematically applying and comparing multiple machine learning algorithms. Among the nine algorithms evaluated, Random Forest demonstrated the highest predictive accuracy, underscoring its suitability for modeling PEBI data. These analyses enrich the literature on the application of machine learning for predicting PEBI and provide a valuable methodological reference for sustainability-related behavioral research. Second, by identifying the relative importance of 20 predictors and examining their heterogeneity across different subsamples, this study advances understanding of the key drivers of PEBI. Third, this research offers new insights into PEBI by exploring interaction patterns among predictors using the SHAP method. The results suggest potential interaction relationships among predictors, illuminating the complex dynamics underlying PEBI. Overall, this study contributes to the existing research paradigm on PEBI by incorporating machine learning techniques and offers novel perspectives for understanding the determinants of PEBI.

## Literature review

### Pro-environmental behavior

As a type of pro-social behavior, PEB plays a crucial role in mitigating environmental degradation and promoting ecological sustainability. Despite its recognized importance, a unified definition of PEB is still lacking in the existing literature. Various related terms have been used to describe human behaviors that affect the natural environment, such as green behavior^15^, ecological behavior^16^, environmentally friendly behavior^17^, environmentally responsible behavior^18^, environmentally sustainable behavior^19^, eco-friendly behavior^20^, etc. Although these terms may vary in their emphasis, they generally share common activities that support environmental sustainability and reduce ecological harm. Given that this study focuses specifically on individual-level environmental behaviors, PEB is regarded as actions aimed at minimizing the harm of one’s activities on the natural environment^21^. Considering that behavioral intention is a key driver of actual behavior, a growing body of research has used PEBI as a proxy for PEB. Accordingly, this study focuses on PEBI to better understand the factors that stimulate individuals’ environmental intentions. In the following section, the influencing factors of PEBI that have been discussed in the existing literature are reviewed.

### Antecedents of pro-environmental behavior intention

Existing research has extensively explored various influential factors that may trigger PEBI. Guided by previous literature and TPB^5,12^, we posit that the main factors influencing PEBI can be categorized into four categories: environmental mindset, social-ecological interaction, well-being, and demographic factors. TPB is a prevalent theory and served as the basis for many studies related to pro-environmental behavior^22^. According to TPB, an individual’s behavior depends on behavioral intention, which was predicted by their attitudes toward the behavior, subjective norm, and perceived behavioral control^5^. This perspective implies that behavioral intentions are shaped by personal determinants, social environment, and non-volitional factors^23^. To enhance its explanatory and predictive effectiveness in the environmental domain, researchers have extended the model by incorporating additional variables such as environmental concern^24^, sense of responsibility^25^, and emotion^26^. These extensions provide a theoretical insight for integrating multiple factors as antecedents of PEBI. Thus, our study is based on the TPB, with additional variables included to predict individuals’ PEBI.

**Environmental mindset.** An environmental mindset reflects individuals’ perceptions and evaluations of the natural environment. From the perspective of TPB, individuals’ intentions to engage in specific behaviors are influenced by their evaluations of those behaviors^5^, providing a theoretical basis for linking environmental mindset to PEBI. Empirical studies support the relationship. For example, Liu et al.^27^ found that environmental knowledge enhances environmental attitudes, which in turn significantly promote environmental behavioral intentions and subsequent behaviors. In addition, recent research has shown that climate change health risk perception can also influence personal PEBI and PEB^6^. Taken together, these findings suggest that environmental mindset plays a critical role in shaping individuals’ PEBI.

**Social-ecological interaction**. The category of social-ecological interaction encompasses multiple contextual and relational factors, including social capital, internet use, and individuals’ connection to nature. In particular, existing studies conceptualize social capital along four key dimensions: social trust, social networks, social participation, and social norms^28^. Empirical evidence indicates that these social-ecological interaction factors play an important role in shaping PEBI. For example, Tan et al^29^. found that institutional trust and interpersonal trust significantly and positively affect individuals’ willingness to pay for environmental protection. Similarly, drawing on TPB, recent research revealed that social media use is positively associated with attitude and subjective norm, thereby fostering stronger pro-environmental intentions^23^. In addition, individuals’ connectedness to nature has been shown to significantly predict PEBI^30,31^. Overall, social-ecological interaction serves as an important factor for understanding individuals’ PEBI.

**Well-being**. Personal well-being has been recognized as an important factor influencing PEBI, encompassing aspects such as positive and negative emotions, life satisfaction, and health status^32,33^. From a subjective and psychological well-being perspective, Piao and Managi^33^ found that positive emotions (e.g., pleasure, enjoyment, and smiling) and life satisfaction are positively associated with engagement in PEB. On the contrary, Rees et al.^34^ suggested that anticipated negative emotions arising from unsustainable behavior can strengthen individuals’ environmental behavioral intentions. Taken together, these findings indicate that examining personal well-being provides valuable insights into the determinants of PEBI.

**Demographic characteristics**. Antecedents associated with demographic characteristics have been widely discussed in environmental protection research, including gender, age, income, education level, marital status, and household registration. For instance, Liobikienė and Juknys^35^ reported that women tend to be more environmentally friendly than men are, and that older and more educated individuals are more environmentally friendly. However, Zhao et al.^36^ did not find that gender has a significant impact on environmentally friendly behavior. Similarly, the relationship between income and PEBI remains inconclusive. While Berthold et al.^37^ found a negative association between income and individuals’ willingness to engage in environmental protection, this result was not supported in other studies^38^. Overall, these findings highlight the important role of demographic factors and underscore the need for more comprehensive and nuanced research on PEBI.

### Applications of machine learning

The most commonly employed methods for predicting PEBI in academic research include logistic regression, ordinary least squares (OLS), and structural equation modeling (SEM), among others. For example, Lahoti et al.^39^ utilized a multinomial logistic regression analysis to examine the correlation between respondents’ perceived connection with nature and their engagement in environmental activities. Gong et al.^40^ applied the OLS method to estimate the effect of internet use on individuals’ PEB. Although these analytical tools demonstrate good explanatory power in modeling relationships between variables, their reliance on linear assumptions may limit their ability to capture non-linear interactions in complex scenarios. In contrast, machine learning techniques can offer superior flexibility by effectively capturing both linear and non-linear relationships^41^. This algorithm can handle heterogeneous datasets without strict distributional assumptions, and provide high precision and adaptability through analyzing and interpreting a large-scale dataset.

With the striking upsurge of big data, a few recent studies on environmental activities have increasingly incorporated machine learning methods in academic studies. For example, the random forest model was used to examine the effect of various factors on household waste separation intentions and behaviors^42^. Another study by Rezapouraghdam et al.^43^ adopted the machine learning technique to predict visitors’ green behavior in marine protected areas. More recently, multiple machine learning models were employed to explore the influencing mechanism in the dual pro-environmental intention-behavior gap^3^. These efforts underscore the necessity and urgency of leveraging machine learning methods to predict individuals’ PEBI, as such approach can uncover complex patterns and enhance prediction accuracy. Therefore, this study conducts multiple machine learning algorithms to investigate how the various influencing factors of environmental mindsets, social-ecological interaction, well-being, and demographic characteristics affect individuals’ PEBI. Furthermore, it seeks to evaluate the relative importance of the various variables, offering a comprehensive and data-driven understanding of the determinants of PEBI.

## Data and methods

### Data acquisition and description

The data utilized in the present study were drawn from the 2021 CGSS, a large-scale, nationally representative social survey project initiated in 2003. Conducted by the Survey and Data Center of Renmin University of China, this survey has become one of the most comprehensive and authoritative datasets for understanding the social, economic, and cultural dynamics of Chinese society. By employing a rigorous multistage stratified probability sampling method, the survey captures the diversity and complexity of China’s population across urban and rural areas. The 2021 wave of the CGSS collected data from multiple provinces in mainland China, encompassing a broad range of variables, such as demographic characteristics, socio-economic status, social attitudes, subjective well-being, environmental attitudes, and behavioral intentions. These variables provide comprehensive insights into the interactions between individuals and the environment, making the dataset particularly valuable and well-suited for investigating the multifaceted influences on PEBI.

The 2021 CGSS dataset initially comprised 8,148 respondents. To ensure the reliability of the analysis, a two-step data cleaning procedure was conducted. First, PEBI, the core variable of this study, was used as the primary screening criterion. Observations with missing or invalid responses on the PEBI items were removed, resulting in 2,526 valid cases. Second, the dataset was further screened based on the predictor variables included in the machine learning models. Cases with missing values or invalid responses on any predictor variable were excluded. Invalid responses mainly referred to options such as “do not know,” “unable to choose,” or other non-substantive responses provided by the respondents. After this second round of data cleaning, the final analytical sample consisted of 1,560 valid observations. Regarding sample size, previous studies suggest that in machine learning analyses, the sample size should be at least 10 to 100 times the number of features or attributes included in the model^44^. A total of 20 predictor variables were included in our study; therefore, the final sample size of 1,560 provides an adequate basis for machine learning analysis.

### Ethics approval

The data used in this paper are publicly available data from the Chinese General Social Survey 2021 (http://cgss.ruc.edu.cn). Research ethics approval for data collection in the CGSS dataset was granted by the Institutional Review Board of Renmin University, and the survey was conducted in accordance with the ethical principles outlined in the Declaration of Helsinki. All participants provided written informed consent before taking part in the survey. As this paper uses a public database, no additional ethical approval was required.

### Variables and measurement

PEBI served as the dependent variable and was measured using a 5-point Likert scale ranging from 1= “very unwilling” to 5 = “very willing”. Participants were asked to evaluate their level of willingness to engage in the following activities: (1) Regularly sorting household waste for proper disposal; (2) Recycling and reusing household items; (3) Engaging in discussions with other residents to develop waste sorting plans; (4) Volunteering regularly to participate in public service activities aimed at maintaining environmental cleanliness. Notably, the CGSS2021 dataset contains only a limited number of items that directly capture individuals’ PEBI. These selected items reflect observable pro-environmental intention and are considered representative indicators of environmental engagement willingness. Following the existing literature^45^, a composite score was first calculated by taking the mean value of the four items. To facilitate the application of classification-based machine learning models, the continuous PEBI score was subsequently transformed into a binary variable using the sample mean as the threshold. Specifically, respondents with a score greater than or equal to the mean were coded as 1, representing individuals exhibiting PEBI, while those below the mean were coded as 0, reflecting the absence of such intentions.

With regard to independent variables, with reference to prior literature^12,46^, our study identified 20 key predictive variables, which were divided into four categories: environmental mindsets, social-ecological interaction, well-being, and demographic characteristics. Detailed information about the measurement and coding of variables is presented in Table 1. For predictors measured using multiple survey items, composite scores were calculated by taking the mean value of the corresponding items. To ensure comparability across variables, all variables were subsequently normalized using min-max scaling (see Eq. 1) prior to model estimation.

1 $$X=\frac{{{X_i} - {X_{\hbox{min} }}}}{{{X_{\hbox{max} }} - {X_{\hbox{min} }}}}$$

where *X*_*i*_ represents the original value of the feature, *X*_*min*_ and *X*_*max*_ denote the minimum and maximum values of the feature, respectively, and *X* is the normalized value.

**Table 1:** Content interpretation, measurement, and sources of variables.

Variables	Item Content and measurement	Source
PEBI	To what extent are you willing to make the following efforts to address the challenges of waste management? (a) Regularly sort household waste for proper disposal; (b) Recycle and reuse household items; (c) Engage in discussions with other residents to develop waste sorting plans; (d) Volunteer regularly to participate in public service activities aimed at maintaining environmental cleanliness. (1 = Not at all willing; 5 = Very willing)	Wu and Zhang^47^
Environmental attitude	(a) To protect the environment, are you willing to pay higher prices? (b) To protect the environment, are you willing to pay higher taxes? (c) To protect the environment, are you willing to lower your standard of living? (1 = Extremely unwilling to; 5 = Extremely willing to)	Wang et al.^48^
Environmental responsibility	To what extent do you agree with the following statements? (a) It is difficult for me to do anything for environmental protection; (b) There are more important things in life than environmental protection; (c) Unless all people do otherwise, if I protect the environment, it is meaningless. (1 = Strongly agree; 5 = Strongly disagree)	Zhang et al.^49^
Environmental concern	Generally speaking, how concerned are you about environmental problems? (1 = Not at all concerned; 5 = Extremely concerned)	Zhang et al.^49^
Environmental knowledge	(a) To what extent do you think air pollution caused by vehicle emissions is harmful to the environment? (b) To what extent do you think air pollution caused by industrial exhaust gas is harmful to the environment? (c) To what extent do you think the use of pesticides and fertilizers in agricultural production is harmful to the environment? (d) To what extent do you think pollution in China’s rivers, lakes, and other bodies of water is harmful to the environment? (1 = Not harmful at all; 5 = Extremely harmful to the environment)	Zhang et al.^49^
Environmental risk perception	To what extent do you think the following environmental issues are serious in your local area? Air pollution; Water pollution; Soil pollution; Noise pollution; Industrial waste pollution; Household waste pollution. (1 = No problem; 6 = Very serious)	Yang et al.^50^
Government performance perception	(a) How do you think the local government has done in environmental protection over the past five years? (b) How do you think the central government has done in environmental protection over the past five years? (1 = Attention was one-sided to economic development and environmental protection work was neglected; 5 = Great achievements)	Cheng et al.^51^
Social trust	Generally speaking, do you agree that most people in society are trustworthy? (1 = Strongly disagree; 5 = Strongly agree)	Miao et al.^52^
Social participation	Did you vote in the last neighborhood/village election? (1 = No; 2 = Yes)	Wan and Du^28^
Social network	(a) What is the frequency of your social activities with your neighbors (e.g., hanging out, watching TV, eating, playing cards, etc.)? (b) What is the frequency of social activities with other friends (e.g., hanging out, watching TV, eating, playing cards, etc.)? (1 = Never; 7 = Almost daily)	Wan and Du^28^
Internet use	How often did you use the Internet (including mobile Internet) in the past year? (1 = never; 5 = very frequently)	Miao et al.^52^
Connection to nature	If you can, how much do you like to go out into nature and do outdoor activities? (1 = not at all; 5 = very much)	Nisbet et al.^53^
Happiness	Overall, do you feel happy with your life? (1 = Very unhappy; 5 = Very happy)	Shen et al.^54^
Negative emotion	In the past four weeks, how often have you felt depressed or upset? (1 = Never; 5 = Always)	Zhao and Huang^55^
Health status	How do you perceive your current physical health status? (1 = Very unhealthy; 5 = Very healthy)	Zhang et al.^56^
Age	What is your date of birth?	Yang et al.^50^
Gender	What is your gender? (1 = Male; 2 = Female)	Yang et al.^50^
Income	What was your personal total income last year?	Zhang et al.^49^
Education	What is your current highest level of education? (1 = No education; 13 = Graduate degree or above)	Yang et al.^50^
Marital status	What is your current marital status? (1 = Otherwise; 2 = Married)	Wang et al.^48^
Household registration	What is your current household registration status? (1 = Agricultural type; 2 = Non-agricultural one)	Zhang et al.^57^

In addition, we present the descriptive statistics and variance inflation factors (VIF) analysis for all variables. As indicated in Table 2, the values of all variables are below 3, indicating no serious multicollinearity among the variables and ensuring the reliability of the dataset for subsequent analyses. In terms of demographic characteristics, the sample comprises 49.6% males and 50.4% females, and 71.5% of the participants were married. Regarding household registration, 64.6% of participants hold agricultural household registration, while 35.4% hold non-agricultural household registration.

**Table 2:** Descriptive statistics of the variables.

Variable content	Obs	Mean	SD	Min	Max	VIF
Dependent variable						
PEBI	1560	3.982	0.739	1	5	N
**Environmental mindsets**						
Environmental attitude	1560	3.064	0.902	1	5	1.175
Environmental responsibility	1560	2.781	0.779	1	5	1.154
Environmental concern	1560	3.635	0.883	1	5	1.162
Environmental knowledge	1560	3.629	0.644	1	5	1.077
Environmental risk perception	1560	3.104	1.139	1	6	1.210
Government performance perception	1560	3.892	0.811	1	5	1.164
**Social-ecological interaction**						
Social trust	1560	3.709	0.921	1	5	1.101
Social participation	1560	1.521	0.500	1	2	1.117
Social network	1560	3.886	1.714	1	7	1.048
Internet use	1560	3.564	1.600	1	5	1.850
Connection to nature	1560	3.313	1.172	1	5	1.112
**Well-being**						
Happiness	1560	3.999	0.763	1	5	1.229
Negative emotion	1560	2.029	1.038	1	5	1.361
Health status	1560	3.621	1.033	1	5	1.515
**Demographic factors**						
Age	1560	50.067	17.156	18	94	2.199
Gender	1560	1.504	0.500	1	2	1.052
Income	1560	57294.235	362595.021	0	9,993,000	1.015
Education	1560	5.619	3.331	1	13	1.938
Marital status	1560	1.715	0.451	1	2	1.094
Household registration	1560	1.354	0.478	1	2	1.309

### Research framework

**Fig. 1:**
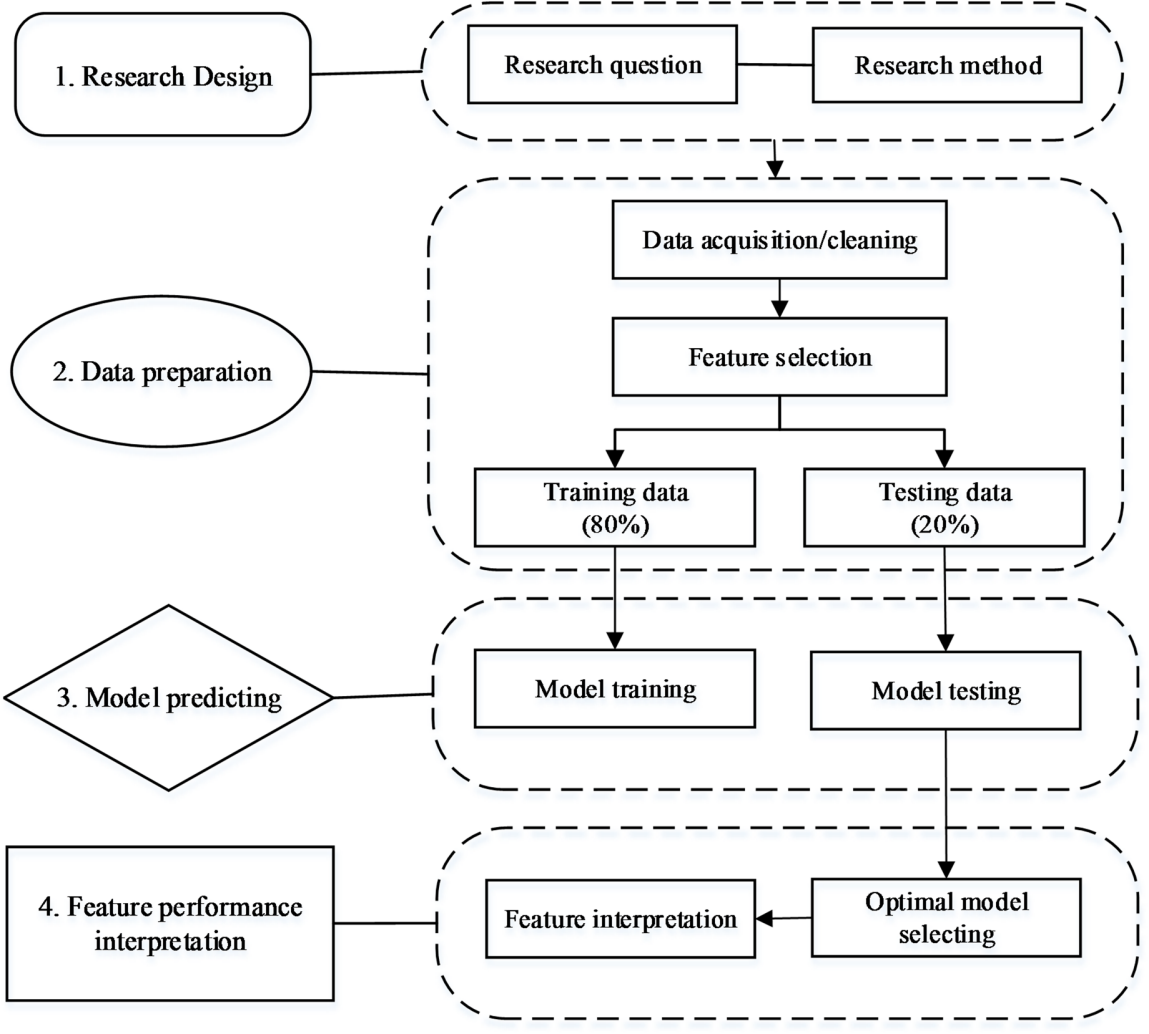
The research framework of PEBI analysis.

To systematically predict and interpret the determinants of PEBI, this study adopted a four-stage research procedure grounded in machine learning literature^58^. As illustrated in Fig. 1, this structured framework ensures both analytical rigor and interpretability.

*Step 1: Research design.* Drawing upon extensive literature on PEBI, this study formulated the core research question: to what extent can machine learning techniques accurately predict individuals’ PEBI, and which factors play the most significant roles in this prediction? Clarifying the research question at the outset enabled us to align subsequent data preparation and model analysis with the overall research objectives.

*Step 2: Data preparation.* Data were obtained from the 2021 wave of the Chinese General Social Survey (CGSS), a nationally representative dataset. After removing observations with missing or invalid values, a total of 20 variables were selected as model inputs to investigate both prediction accuracy and feature contribution. Variance inflation factor (VIF) diagnostics were conducted to ensure the absence of problematic multicollinearity. The final dataset was randomly partitioned into a training set (80%) and a testing set (20%) using Python, providing the groundwork for predictive analysis.

*Step 3: Model predicting.* We employed nine widely used machine learning algorithms to predict individuals’ PEBI in the model-predicting step. GridSearchCV was used to tune the models’ hyperparameters. In addition, K-fold cross-validation was employed to measure the accuracy of the machine learning models.

*Step 4: Feature performance interpretation.* The final step focused on model selection and interpretation. Based on the best-performing model, the SHAP method was applied to rank all features according to their contributions, thereby identifying the most influential factors driving individuals’ PEBI. Next, SHAP values were examined across subgroups (e.g., gender and age) to explore heterogeneity in feature effects. In addition, SHAP interaction values were used to assess potential interaction effects between key predictors.

### Machine learning methodology

Machine learning approaches offer distinct advantages in capturing complex nonlinear relationships and predicting unobserved data, rendering them useful for solving real-world problems^59,60^. Given that our study focuses on a classification problem in the context of PEBI, we implemented multiple ensemble and individual machine learning algorithms. Specifically, ensemble methods (i.e., eXtreme Gradient Boosting (XGBoost), Gradient Boosting, LightGBM, and Random Forest) were selected for their ability to combine multiple base learners using a divide-and-conquer approach, which improves predictive performance^61^. For example, Random Forest constructs a multitude of decision trees through combining different sets of features and samples, thereby enhancing predictive accuracy and model stability^62^. XGBoost incorporates an optimized tree-learning algorithm with integrated regularization to efficiently process sparse data and distributed computation, thereby achieving superior resource efficiency^63^. To explore the best-performing prediction of PEBI, we also included five individual machine learning algorithms, including Support Vector Machine (SVM), Decision Tree, K-Nearest Neighbors (KNN), Logistic Regression, and Multi-Layer Perceptron (MLP). These representative algorithms have been applied in prior studies addressing classification tasks, allowing for a comparison of predictive performance across models. For example, Zhang et al.^64^ compared seven algorithms—including SVM, MLP, KNN, XGBoost, Random Forest, AdaBoost, and Decision Tree—for predicting CSR ratings, while Choudhury et al.^45^ applied multiple machine learning techniques, such as Decision Tree, Random Forest, Gradient Boosting, XGBoost, KNN, and SVM, to a binary classification problem in the context of green purchase behavior. Overall, machine learning models have demonstrated superior predictive performance, particularly for large and complex datasets; thus, we adopted them to predict individuals’ PEBI in the present work.

## Results

### Model evaluation criterion

To evaluate model performance and guide model selection, we employed five commonly used evaluation metrics in classification tasks, including accuracy, recall, precision, F1 score, and area under the curve (AUC). These metrics capture model performance from multiple perspectives, reflecting the predictive accuracy and reliability of the model. As for the four data categories, true positive (TP) refers to cases where individuals intend to perform PEB and are correctly predicted as such, whereas true negative (TN) denotes cases where individuals do not have PEBI and are correctly predicted as non-intenders. False positive (FP) represents cases where individuals do not have PEBI but are predicted as having such intention, while false negative (FN) represents cases where individuals have PEBI but are predicted as not having such intention.

Accuracy is one of the most intuitive evaluation metrics (see Eq. 2), reflecting the proportion of correct predictions by a machine learning model.

2 $$Accuracy=\frac{{TP+TN}}{{TP+TN+FP+FN}}$$

Recall measures the proportion of actual PEBI cases that are accurately detected by the model to the total number of PEBI cases (see Eq. 3), highlighting its effectiveness in capturing positive instances.

3 $$Recall=\frac{{TP}}{{TP+FN}}$$

Precision refers to the proportion of correctly predicted positive cases among all cases predicted as positive (see Eq. 4), reflecting the reliability of positive predictions.

4 $$Precision=\frac{{TP}}{{TP+FP}}$$

F1 value represents the harmonic mean of precision and recall (see Eq. 5), providing a balanced measure of precision and recall.

5 $$F1score=\frac{{2 * Precision * Recall}}{{Precision+Recall}}$$

AUC is the area under the receiver operating characteristic (ROC) curve (see Eqs. 6 and 7), with higher values indicating better classification performance of the machine learning model. An AUC greater than 0.5 suggests the model performs better predictions.

6 $$TPR=\frac{{TP}}{{TP+FN}}$$

7 $$FPR=\frac{{FP}}{{TN+FP}}$$

### Results of predictive modeling

We compared the predictive performance of nine different machine learning algorithms, including XGBoost, LightGBM, Random Forest, Gradient Boosting, Logistic Regression, SVM, Decision Tree, MLP, and KNN. Model performance can vary with different hyperparameter settings^65^. Accordingly, we first employed a grid search to define the optimal ranges of key hyperparameters for each model. Subsequently, five-fold cross-validation was used to select the optimal hyperparameter combinations. Detailed information on hyperparameter tuning for all nine models is provided in Appendix A.

**Fig. 2:**
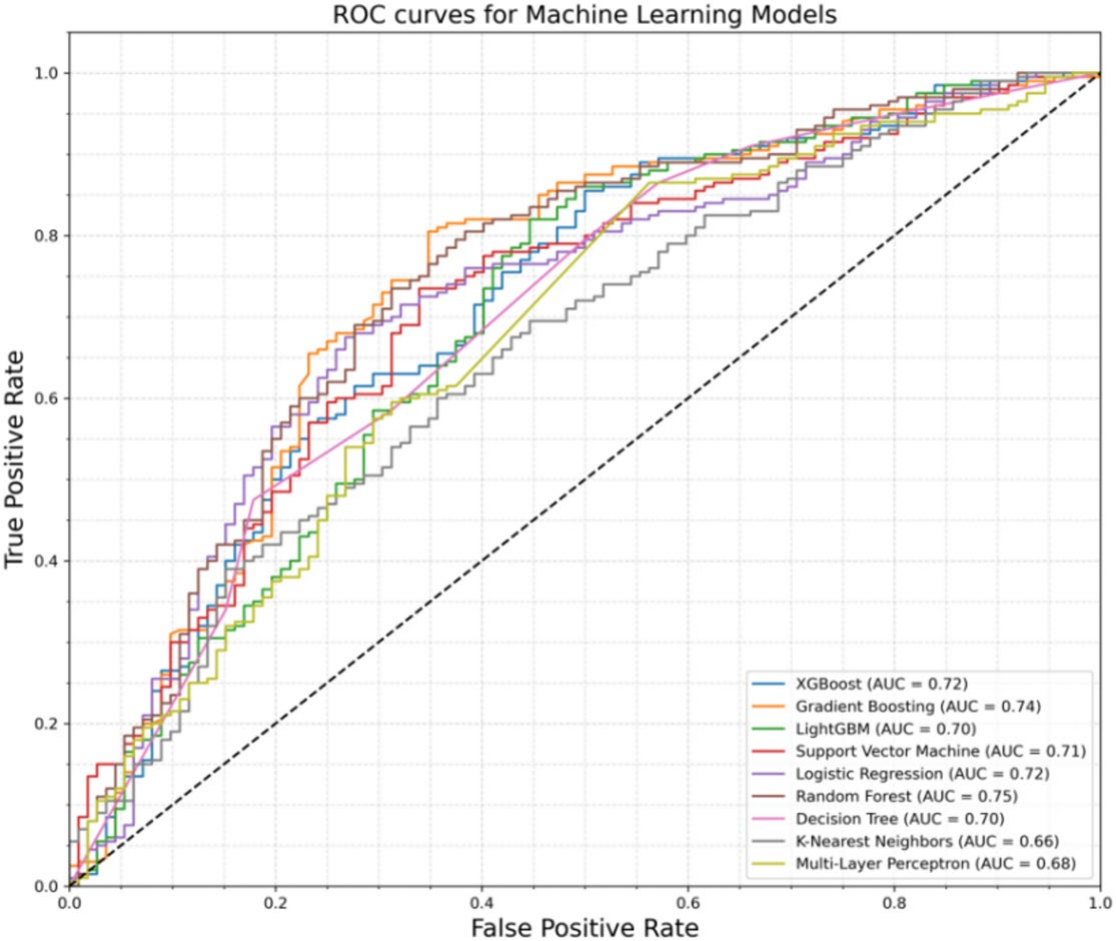
ROC for 9 machine learning models.

Table 3 presents the predictive performance of nine machine learning algorithms in this study. Random Forest model achieved the highest overall accuracy of 0.721, suggesting that the model correctly predicted individuals’ PEBI in 72.1% of cases. For F1 score, precision, and AUC, the Random Forest model again tops the list (F1 = 0.804; Precision = 0.733; AUC = 0.745). In terms of recall, XGBoost performs best, followed closely by LightGBM, while Random Forest achieves comparable results (Recall = 0.890). In addition, Fig. 2 depicts the ROC curves and corresponding AUC value for nine machine learning algorithms. The x-axis represents the false positive rate, while the y-axis represents the true positive rate. A curve approaching the top-left corner of the plot, along with a higher AUC value, indicates better overall model performance. As illustrated, the ROC curve for the Random Forest model lies close to the top-left corner and exhibits the highest AUC value among all models evaluated. Taken together, considering multiple evaluation metrics, the Random Forest model can be considered the most appropriate and effective machine learning approach for predicting individuals’ PEBI for the given scenario.

**Table 3:** Comparison of the prediction performance.

Model	Accuracy	Recall	F1	Precision	AUC
Random Forest	0.721	0.890	0.804	0.733	0.745
XGBoost	0.699	0.920	0.797	0.702	0.716
LightGBM	0.689	0.915	0.790	0.696	0.700
Gradient Boosting	0.696	0.910	0.793	0.703	0.742
Logistic Regression	0.686	0.805	0.767	0.732	0.719
Support Vector Machine	0.686	0.865	0.779	0.709	0.715
Decision Tree	0.708	0.865	0.792	0.730	0.700
Multi-Layer Perceptron	0.699	0.870	0.787	0.719	0.677
K-Nearest Neighbors	0.651	0.775	0.740	0.708	0.661

### Interpretation of feature importance

#### Integral interpretation of feature importance

To investigate the underlying determinants of PEBI, we further integrated the optimal predictive method – Random Forest – with Shapley Additive exPlanations (SHAP) to construct an interpretable machine learning framework. SHAP offers a unified approach to feature attribution grounded in cooperative game theory, where each feature is conceptualized as a player contributing to the model’s prediction^66^. Shapley values assign a unique and fair distribution among all input features, thus quantifying each feature’s marginal contribution. Given its advantages in assessing the relative importance of each feature variable, the SHAP method has been increasingly adopted in empirical studies across diverse domains, such as corporate violation prediction^67^, service satisfaction modeling^58^, and corporate social responsibility performance assessment^64^. Following prior research, we calculated the SHAP value of each feature, as shown in Eq. 7.

7 $$SHAPvalu{e_i}=\sum\limits_{{S \subseteq F\backslash \left\{ i \right\}}} {\frac{{\left| S \right|!(\left| F \right| - \left| S \right| - 1)!}}{{\left| F \right|!}}} \left[ {{f_{S \cup \left\{ i \right\}}}\left( {{x_{S \cup \left\{ i \right\}}}} \right) - {f_S}\left( {{x_S}} \right)} \right]$$

where i is an input feature. *F* is the set of all input features. And *S* is a subset of *F*. $\frac{{\left| S \right|!(\left| F \right| - \left| S \right| - 1)!}}{{\left| F \right|!}}$ is the weight of the subset *S*. Model${f_{S \cup \left\{ i \right\}}}$ is trained with that feature *S* plus feature i. Model${f_S}$ is trained with the feature without feature i. Where ${x_S}$ delegates the values of the input features in the set *S*.

**Fig. 3:**
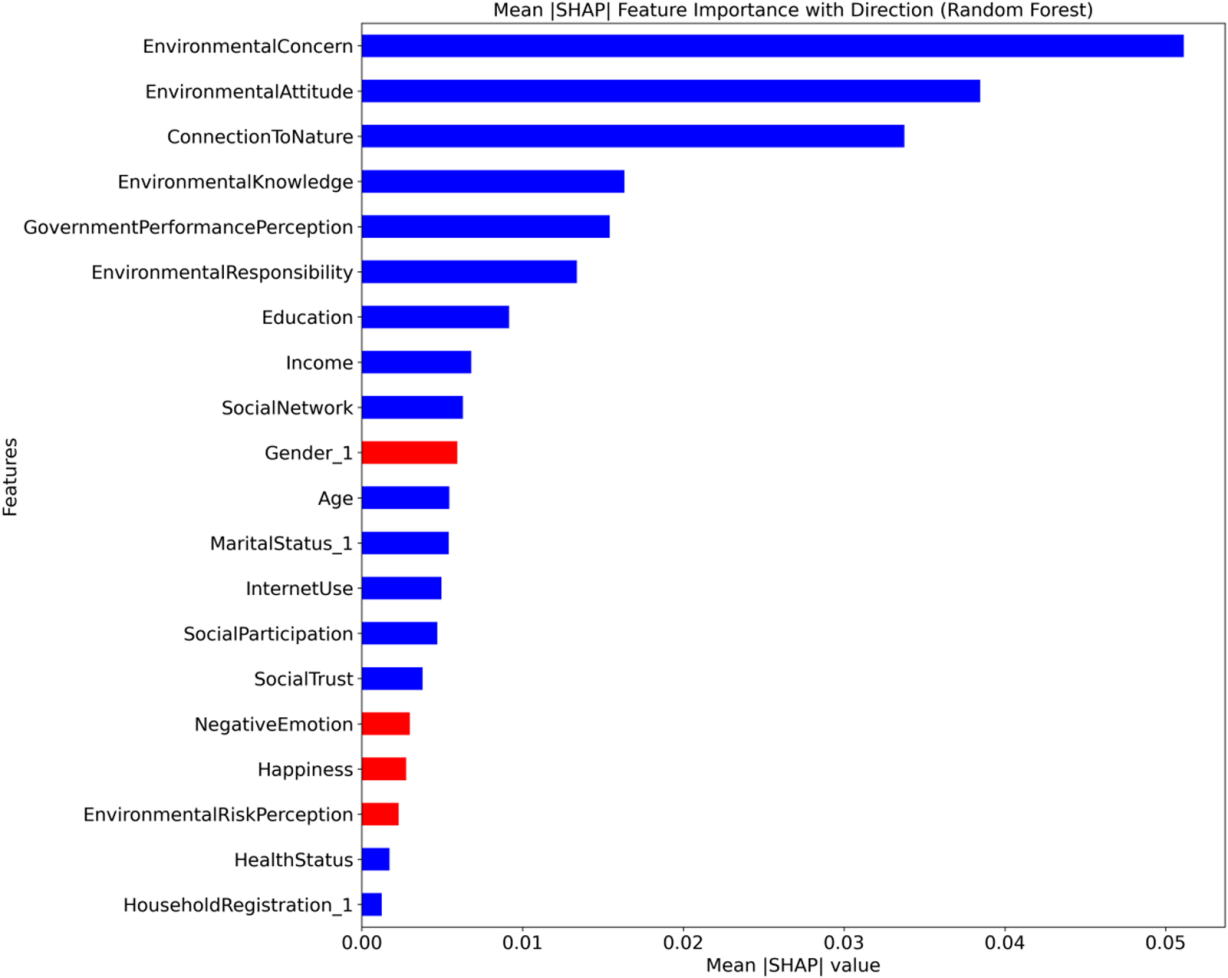
Feature performance ranking based on Random Forest model.

As illustrated in Fig. 3, the SHAP analysis offers a comprehensive visualization of each feature’ s contribution to the prediction of PEBI. Specifically, the horizontal axis represents the mean absolute SHAP values, while the vertical axis shows all input features. A higher mean absolute SHAP value denotes a greater contribution to the predictive outcome. The results reveal that environmental concern is the most influential feature in the Random Forest model, followed by environmental attitude, connection to nature, environmental knowledge, government performance perception, and environmental responsibility. These findings suggest that environmental mindset variables are prominently represented among the top-ranking predictors in explaining individuals’ PEBI within the present analytical framework. Among the subsequent factors, several social-ecological interaction and demographic characteristic factors—such as education, income, and social network—also exhibit relatively high mean absolute SHAP values. Figure 3 further illustrates the positive and negative associations between predictive variables and PEBI, where blue indicates positive contributions and red indicates negative contributions. The results demonstrate that the six most important factors (i.e., environmental concern, environmental attitude, connection to nature, environmental knowledge, government performance perception, and environmental responsibility) are positively related to individuals’ PEBI. By contrast, gender, negative emotion, happiness, and environmental risk perception are negatively associated with individuals’ PEBI.

### Heterogeneous interpretation of feature importance

Given its superior predictive performance for PEBI, the Random Forest model was further employed to conduct a heterogeneity analysis aimed at uncovering the differences in feature contributions across subgroups. Table 4 presents both the mean absolute SHAP values and the effects (i.e., the sum of SHAP values for a given feature across all samples) of the predictors for the male and female subsamples. The comparative results indicate the distinct feature influences between the two groups. The results show that environmental attitude emerges as the most influential predictor in both subsamples. Among subsequent environmental mindset factors, environmental concern, environmental responsibility, and environmental knowledge also play relatively important roles in the male subsample, whereas government performance perception and environmental concern appear more prominent in the female subsample. The aforementioned influential predictors are positively associated with individuals’ PEBI. Regarding social-ecological interaction factors, connection to nature demonstrates an important contribution across both groups, highlighting the role of individuals’ emotional and cognitive bonds with the natural environment in promoting PEBI. In terms of demographic factors, age and income display divergent contribution patterns between the two subsamples. Specifically, income exhibits a negative contribution to the prediction of PEBI among male respondents, whereas age shows a negative contribution in the female subsample.

**Table 4:** Feature performance differences based on male and female samples.

Feature	Feature of male		Feature of female	
	Mean absolute SHAP value	Effect	Mean absolute SHAP value	Effect
**Environmental mindsets**				
Environmental attitude	0.057	8.547	0.041	6.482
Environmental responsibility	0.039	6.090	0.004	−0.651
Environmental concern	0.044	6.868	0.036	5.653
Environmental knowledge	0.022	3.410	0.012	1.920
Environmental risk perception	0.003	−0.247	0.007	−1.127
Government performance perception	0.004	0.440	0.036	5.709
**Social-ecological interaction**				
Social trust	0.003	0.328	0.002	−0.159
Social participation	0.009	1.411	0.002	−0.001
Social network	0.006	0.929	0.005	0.692
Internet use	0.009	1.375	0.014	2.285
Connection to nature	0.029	3.981	0.038	5.567
**Well-being**				
Happiness	0.002	−0.006	0.008	1.082
Negative emotion	0.009	−1.087	0.006	−1.026
Health status	0.002	−0.305	0.009	1.396
**Demographic factors**				
Age	0.018	2.810	0.027	−4.323
Income	0.011	−1.723	0.003	0.395
Education	0.011	1.651	0.020	3.167
Marital status	0.003	0.403	0.000	−0.022
Household registration	0.001	0.059	0.001	0.133

Considering that individuals’ age may influence the relationship between predictive variables and PEBI^68^, we further examined the age-based heterogeneity in feature performance. Following prior research^69^, individuals under the age of 45 were categorized as the younger group, while those aged 45 and above were designated as the older group. Table 5 presents the feature importance and effects across the two groups. Specifically, in the older group, environmental attitude ranks as the most important feature, followed by environmental concern, education, and connection to nature. In the younger group, environmental attitude also emerged as the influential factor for predicting PEBI, exhibiting the highest mean absolute SHAP value. The subsequent important predictors include connection to nature, environmental concern, and environmental knowledge. The aforementioned influential predictors are positively related to PEBI. Furthermore, the age-related differences were observed in terms of negatively associated features. Among older individuals, negative emotion and gender show negative contributions to the prediction of PEBI, whereas income, environmental risk perception, and happiness exhibit negative contributions in the younger group.

**Table 5:** Feature performance differences based on older and younger samples.

Feature	Feature of older		Feature of younger	
	Mean absolute SHAP value	Effect	Mean absolute SHAP value	Effect
**Environmental mindsets**				
Environmental attitude	0.063	11.816	0.057	6.710
Environmental responsibility	0.010	1.164	0.017	1.971
Environmental concern	0.052	9.968	0.038	4.542
Environmental knowledge	0.017	3.012	0.033	3.964
Environmental risk perception	0.004	0.304	0.011	−0.010
Government performance perception	0.019	3.681	0.011	1.335
**Social-ecological interaction**				
Social trust	0.005	0.838	0.006	0.714
Social participation	0.005	1.003	0.005	0.001
Social network	0.010	1.917	0.019	2.244
Internet use	0.011	1.192	0.002	0.244
Connection to nature	0.033	5.813	0.041	4.737
**Well-being**				
Happiness	0.003	0.149	0.004	−0.352
Negative emotion	0.004	−0.641	0.004	0.523
Health status	0.003	0.504	0.003	0.291
**Demographic factors**				
Gender	0.020	−0.340	0.007	0.897
Income	0.013	2.581	0.028	−3.312
Education	0.050	9.683	0.009	1.032
Marital status	0.004	0.763	0.004	0.521
Household registration	0.003	0.136	0.007	0.250

### Interaction effects between features

**Fig. 4:**
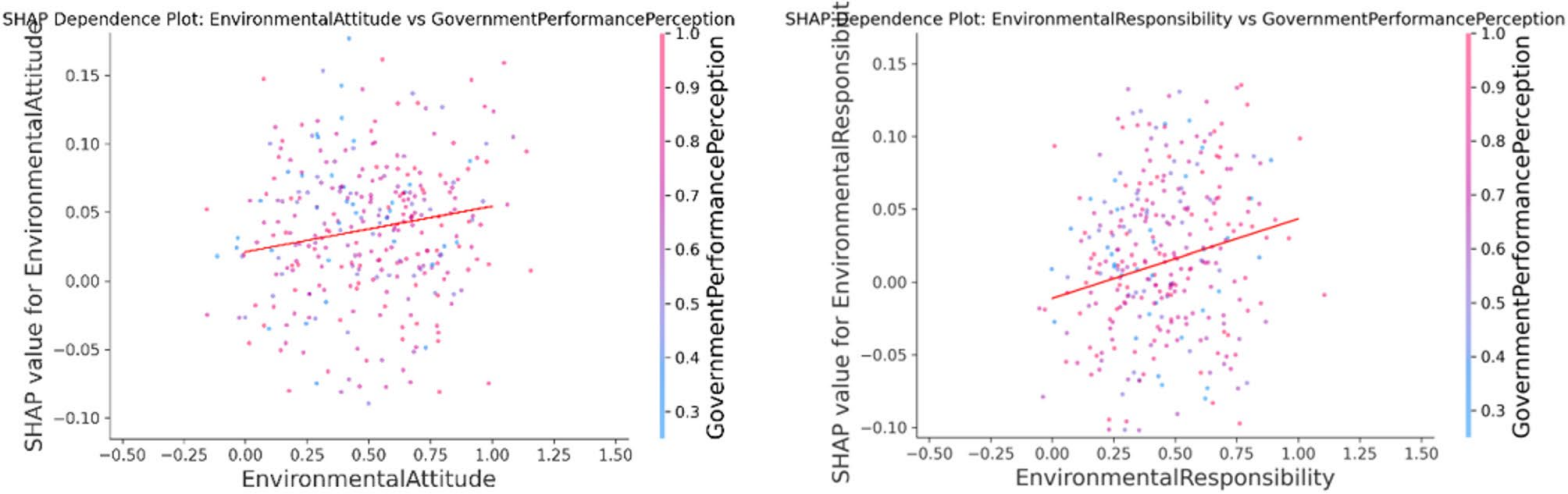
Interaction effects between features. (**a**) Interaction between environmental attitude and government performance perception. (**b**) Interaction between environmental responsibility and government performance perception.

To obtain deeper insights into the multi-dimensional relationships among predictors, we further investigated the effects of feature interactions on individuals’ PEBI using SHAP analysis. Figure 4 presents the results of interaction effects between features. In the dependence plots, the horizontal axis denotes the selected feature under investigation, while the left vertical axis represents the corresponding SHAP values, indicating the feature’s contribution to the model’s prediction. The right vertical axis displays the interacting features, with color gradients (from blue to red) representing the values of the interacting variables. To illustrate interaction patterns among key predictors, we selectively tested two groups of feature interactions involving environmental mindset variables. As shown in Fig. 4, as environmental attitude and environmental responsibility increase, their SHAP values also increase, indicating that environmental attitude and environmental responsibility are positively associated with PEBI. Further, as government performance perception increases (changing from blue to red), the SHAP values of environmental attitude and environmental responsibility tend to be higher. This indicates that, to some extent, the positive contributions of environmental attitude and environmental responsibility to PEBI are stronger among individuals with higher levels of government performance perception.

## Conclusion and discussion

Although PEBI has been widely studied, the present study aims to provide a more comprehensive understanding of its predictors. Based on nine machine learning algorithms, we investigate the influence of 20 variables on PEBI across four aspects: environmental mindsets, social-ecological interaction, well-being, and demographic characteristics. The results reveal that the Random Forest model outperforms other algorithms, achieving the highest predictive accuracy for PEBI. Furthermore, using the interpretable Random Forest model in combination with SHAP analysis, we found that environmental concern emerges as the top contributor, followed by environmental attitude, connection to nature, environmental knowledge, government performance perception, and environmental responsibility. These findings are consistent with previous research^12^, as the TPB suggests that an individual’s engagement in a behavior is influenced by their evaluative beliefs regarding the behavior^5,6^. Among social-ecological interaction factors, connection to nature was identified as the important contributor in the prediction of PEBI, which supports the previous literature^31^. One possible explanation is that greater engagement with nature may cultivate empathy toward the natural world, which, in turn, could encourage individuals to engage in more PEB^70^. Regarding demographic characteristics, education, and income contributed positively to the prediction of PEBI. In line with prior research, educational attainment is generally positively associated with individuals’ engagement in pro-environmental behaviors^33^, possibly because higher education levels may enhance understanding of environmental issues, which in turn can foster pro-environmental intentions. Further, our study investigates the heterogeneity of feature importance across gender and age subgroups. The results demonstrate that environmental attitude exhibits the highest predictive power across all subsamples, underscoring the pivotal role of environmental attitude in promoting PEBI.

Finally, we explored potential interaction effects among environmental mindset variables. The results indicated that, to some extent, the interaction effects exist between environmental attitude, environmental responsibility, and government performance perception. Government performance perception reflects individuals’ subjective evaluation of the government actions in environmental governance, capturing how citizens interpret and internalize policy initiatives^51^. Drawing on government-citizen interaction theory, effective environmental governance relies on public acknowledgment of governmental legitimacy, reciprocal responsiveness, and trust established between actors and citizens^71,72^. From this perspective, a higher perception of government performance conveys the signal that the government is actively fulfilling its environmental responsibilities^73^. Such supportive signals may enhance individuals’ trust and perceived legitimacy, thereby strengthening the translation of environmental attitude and sense of responsibility into PEBI. Supporting this argument, Kulin and Johansson Sevä^74^ found that environmentally concerned individuals are more likely to engage in public pro-environmental behavior in countries with fair, effective, and impartial government institutions. Overall, these findings highlight that PEBI is shaped by multiple factors, emphasizing the complex and multifaceted nature of environmental action.

### Theoretical implications

The present study offers several important theoretical implications. First, this study contributes to PEBI literature by applying machine learning methods to predict individuals’ PEBI. The existing research in this field has predominantly relied on traditional regression models, which may be constrained by assumptions of linear relationships, error distributions, and multicollinearity. In contrast, our study adopts the machine learning methods that are more flexible and data-driven, enabling the identification of complex relationships among variables^13^. In particular, by evaluating and comparing the predictive performance of nine machine learning algorithms, Random Forest was identified as the top-performing model among those evaluated, achieving the highest predictive accuracy for PEBI. Moreover, based on the Random Forest model and SHAP analysis, we develop an interpretable model that provides novel insights into the key drivers of PEBI based on the current dataset. Thus, this study demonstrates the effectiveness of interpretable machine learning in sustainability-related behavioral research, thereby offering a methodological reference for future studies seeking to apply novel predictive methods.

Second, our study extends the research paradigm of existing PEBI literature and contributes to TPB by constructing a multidimensional predictive framework for PEBI. Prior studies on the determinants of PEBI have typically adopted a relatively narrow perspective, focusing on a limited set of predictors. In contrast, our research develops an integrative model that simultaneously examines the effects of environmental mindsets, social-ecological interaction, well-being, and demographic factors on individuals’ PEBI, thereby offering a comprehensive and systematic understanding of its antecedents. Furthermore, by ranking the relative importance of predictors based on the Random Forest model and SHAP analysis, we found that environmental concern and environmental attitude significantly contribute to the prediction of PEBI. These findings provide empirical support for TPB by highlighting that an individual’s engagement in a behavior is influenced by their evaluative judgment regarding the behavior^5,6^. Thus, our study extends and enriches the PEB literature and advances TPB by proposing a comprehensive determinant model for PEBI.

Finally, the present study provides novel insights into PEBI research by investigating the interactive effects among key predictors. While most existing studies employing traditional regression analyses emphasize the independent contributions of predictors on PEBI, they often fall short in capturing the intricate and non-linear interactions. Based on machine learning methods, we incorporated the interaction terms into our research model to uncover the combined effects of variables. Our findings suggest the potential interaction pattern between environmental attitude and responsibility and individuals’ perceptions of government performance. Prior research also provides supporting evidence, suggesting that quality of government can shape the translation of environmental concern into pro-environmental behavior^74^. Overall, by considering the variable interaction, this study offers a more nuanced understanding of the drivers of PEBI.

### Practical implications

Our findings also provide significant practical implications. Firstly, our study highlights that environmental concern and environmental attitude play a critical role in predicting PEBI; therefore, it is crucial to employ strategies that can effectively cultivate and enhance individuals’ environmental mindsets. Related initiatives can be implemented to foster individuals’ evaluative and affective orientations toward the environment. Specifically, community organizations and policymakers can integrate environmental ethics and sustainability topics into community programs through concrete and actionable initiatives. For example, community programs may include waste sorting competitions or environmental knowledge quizzes, with small incentives such as thank-you cards or eco-friendly gifts to encourage participation. Such initiatives aim to foster genuine affect toward PEBI, thereby motivating individuals to engage in environmentally responsible practices actively.

Second, given the important contribution of government performance perception in the prediction of PEBI, it is essential for governments not only to fulfill their environmental responsibilities but also to actively communicate their environmental achievements to the public. Transparent and visible environmental governance can strengthen public trust and encourage stronger engagement in PEB^51^. Practical measures may include establishing official accounts on popular social media platforms (e.g., Weibo, TikTok) to disseminate environmental policy interpretations and display the tangible outcomes of environmental initiatives. These strategies can therefore play a crucial role in promoting individuals’ PEBI.

Third, social ecological interaction factors such as connection to nature and social network also contribute to shaping individuals’ PEBI. Strengthening individuals’ emotional and experiential bonds with the natural environment is therefore essential^75^. Local government and environmental organizations should promote nature-based recreational and educational activities, such as eco-tourism and outdoor environmental education programs, to cultivate a deeper sense of connection to nature and thereby foster individuals’ PEBI. In addition, digital platforms and social media can serve as powerful tools for building pro-environmental communities, encouraging the sharing of environmental knowledge and personal experiences, which further amplify individuals’ pro-environmental motivation and intentions through the mechanisms of social learning and peer influence.

### Limitations and future directions

Our study has several limitations that should be acknowledged when interpreting the findings. Firstly, the data utilized were drawn exclusively from the CGSS, which may limit the generalizability of the results to other cultural contexts. Previous research has indicated that PEBI exhibits cultural specificity and may differ across societies, as cultural values and social norms influence individuals’ environmental actions^76,77^. Therefore, the findings of this study should be interpreted primarily within the Chinese socio-cultural context. Future research is encouraged to replicate and extend this study in diverse national or cross-cultural settings to further examine the robustness and external validity of the results.

Secondly, this study does not focus on a specific type of PEBI. However, different types may be driven by distinct psychological and social determinants. As suggested by Hansmann and Binder^78^, green self-identity is more influential in explaining private-sphere pro-environmental behaviors, whereas prescriptive social norms and environmental knowledge play a more important role in predicting public-sphere pro-environmental behaviors. Future research could therefore classify PEBI into different subtypes (e.g., private-sphere vs. public-sphere behavioral intentions) to further investigate whether the determinants identified in this study exert heterogeneous effects across different types of pro-environmental intentions.

Thirdly, the present study focuses more on identifying the key predictors of PEBI and examining interaction effects among these factors using machine learning models, but it does not explore the underlying mediating mechanisms that these predictors influence PEBI. Future research could incorporate additional psychological factors (e.g., self-identity, self-efficacy, and psychological distance of climate change) to examine potential mediating pathways and provide deeper theoretical insights into the drivers of PEBI.

## Supplementary Information

Below is the link to the electronic supplementary material.

Supplementary Material 1

## Data Availability

The data used in this study are publicly accessible. Please visit http://cgss.ruc.edu.cn to access the data.
