# Supplementary material for: Predicting pro-environmental behavioral intention using interpretable machine learning
Source: Sci Rep. 2026 May 11;16:21533. doi: 10.1038/s41598-026-52271-7 (PMC13350094; doi:10.1038/s41598-026-52271-7)
Supplement: Supplementary file 1 — Supplementary Material 1 [file 41598_2026_52271_MOESM1_ESM.docx]

Appendix A. Hyperparameters for Machine Learning Models in Predicting PEBI

| **Model** | **Hyperparameter** | **Search Space** | **Optimal Value** |
| --- | --- | --- | --- |
| XGBoost | n_estimators | [50, 100, 200, 500] | 100 |
|  | max_depth | [3, 5, 7, 9] | 5 |
|  | learning_rate | [0.01, 0.05, 0.1, 0.2, 0.5] | 0.01 |
|  | min_child_weight | [1, 3, 5] | 1 |
| Gradient Boosting | n_estimators | [50, 100, 200, 500] | 100 |
|  | max_depth | [3, 5, 7, 9] | 3 |
|  | learning_rate | [0.01, 0.05, 0.1, 0.2, 0.5] | 0.01 |
|  | min_samples_split | [2, 5, 10] | 2 |
| LightGBM | n_estimators | [50, 100, 200, 500] | 100 |
|  | max_depth | [3, 5, 7, 9] | 5 |
|  | learning_rate | [0.01, 0.05, 0.1, 0.2, 0.5] | 0.01 |
|  | min_child_samples | [5, 10, 20] | 10 |
| Support Vector Machine | C | [0.1, 0.5, 1, 2, 3] | 0.5 |
|  | kernel | ['rbf', 'linear', 'poly'] | rbf |
| Logistic Regression | C | [0.01, 0.1, 1, 10] | 0.1 |
|  | penalty | ['l1', 'l2'] | l1 |
|  | max_iter | [100, 200, 500, 1000] | 100 |
|  | solver | ['liblinear'] | liblinear |
| Random Forest | n_estimators | [50, 100, 200, 500] | 50 |
|  | max_depth | [3, 5, 7, 9, None] | 7 |
|  | max_features | ['sqrt', 'log2'] | sqrt |
|  | min_samples_split | [2, 5, 7] | 5 |
| Decision Tree | max_depth | [3, 5, 7, 9, 11] | 3 |
|  | max_features | ['sqrt', 'log2', None] | None |
|  | min_samples_split | [2, 5, 7] | 2 |
| K-Nearest Neighbors | n_neighbors | [3, 5, 7, 9, 11] | 11 |
|  | weights | ['uniform', 'distance'] | distance |
|  | metric | ['euclidean', 'manhattan'] | manhattan |
| Multi-Layer Perceptron | hidden_layer_sizes | [(50,), (100,), (150,), (50, 50), (100, 50)] | (100, 50) |
|  | alpha | [0.0001, 0.001, 0.01, 0.1] | 0.1 |
|  | learning_rate_init | [0.001, 0.01, 0.1] | 0.1 |
